# Seroprevalence canine survey for selected vector-borne pathogens and its relationship with poverty in metropolitan Pereira, Colombia, 2020^☆^

**DOI:** 10.1016/j.parepi.2022.e00249

**Published:** 2022-04-01

**Authors:** D. Katterine Bonilla-Aldana, Erwin J. Gutiérrez-Grajales, J. Paola Martínez-Arboleda, María Angelica Reina-Mora, Adrián E. Trejos-Mendoza, Soffia Pérez-Vargas, Lorenzo Valencia-Mejía, Luisa F. Marín-Arboleda, Daniela Osorio-Navia, Mariana Chacón-Peña, Luz Victoria González-Colonia, Jaime A. Cardona-Ospina, Erika Vanessa Jiménez-Posada, Andrés Diaz, Jean Carlos Salazar, Manuel Sierra, Fausto Muñoz-Lara, Lysien I. Zambrano, Eduardo Ramírez-Vallejo, Juan Camilo Álvarez, Ingrid Lorena Jaramillo-Delgado, Samuel Pecho-Silva, Alberto Paniz-Mondolfi, Álvaro A. Faccini-Martínez, Alfonso J. Rodríguez-Morales

**Affiliations:** a Semillero de Investigación en Zoonosis (SIZOO), Fundación Universitaria Autónoma de las Américas, Pereira, Risaralda, Colombia; b Institución Universitaria Visión de las Américas, Pereira, Risaralda, Colombia; c Red Colombiana de Enfermedades Transmitidas por Garrapatas en Pequeños Animales (RECEPA) – Colombian Network of Tick-Borne Diseases in Small Animals (RECEPA), Pereira, Risaralda, Colombia; d San Lucas Centro Veterinario y Diagnóstico, Pereira, Risaralda, Colombia; e Grupo de Investigación Biomedicina, Faculty of Medicine, Fundación Universitaria Autónoma de las Américas, Pereira, Risaralda, Colombia; f Grupo de Investigación en Infecciones Emergentes y Medicina Tropical, Instituto para la Investigación en Ciencias Biomédicas, SCI-HELP, Pereira, Risaralda, Colombia; g Vitalcare, Armenia, Quindío, Colombia; h Unit of Scientific Research, School of Medical, Faculty of Medical Sciences, Universidad Nacional Autónoma de Honduras (UNAH), Tegucigalpa, Honduras; i Department of Internal Medicine, Faculty of Medical Sciences, Universidad Nacional Autónoma de Honduras (UNAH), Tegucigalpa, Honduras; j IPS Cardiológica Eduardo Ramírez, Pereira, Risaralda, Colombia; k Grupo de Investigación One-Health, Departamento de Investigación de Enfermedades Infecciosas en Animales, Centro de Diagnóstico Especializado Testmol, Medellín, Antioquia, Colombia; l Universidad Cientifica del Sur, Lima, Peru; m Hospital Nacional Edgardo Rebagliati Martins, Lima, Peru; n Laboratory of Medical Microbiology, Department of Pathology, Molecular and Cell-based Medicine, The Mount Sinai Hospital-Icahn School of Medicine at Mount Sinai, New York, USA; o Department of Pathology, University of Texas Medical Branch, Galveston, TX, USA; p Department of Internal Medicine, Hospital Escuela, Tegucigalpa, Honduras; q Committee of Tropical Medicine, Zoonoses and Travel Medicine, Asociación Colombiana de Infectología, Bogotá, Colombia

**Keywords:** *Anaplasma phagocytophilum*, *Anaplasma platys*, *Ehrlichia canis*, *Ehrlichia ewingii*, *Dirofilaria immitis*, Tick-borne diseases, Hemothropic pathogens, Canine, Zoonotic, Colombia

## Abstract

**Background:**

Tick-borne diseases (TBD) and dirofilariosis are currently not under surveillance in most Latin American countries. In addition, there is a significant lack of studies describing the current situation in most endemic areas, including Colombia. Therefore, seroprevalence studies are crucial for understanding the epidemiology of these vector-borne diseases.

**Methods:**

A serosurvey for TBD and dirofilariosis among 100 dogs was carried out in the municipality of Pereira, located in the Coffee-Triangle region, Colombia. Samples were tested using a rapid assay test system (SNAP® 4Dx®); based on an enzyme immunoassay technique‚ screening for antibodies to *Anaplasma phagocytophilum/platys* (sensitivity 99.1%)‚ *Borrelia burgdorferi* s.l. (98.8%), and *Ehrlichia canis/ewingii* (96.2%) by using specific antigens and checking for *Dirofilaria immitis* antigen based on specific antibodies (99.2%). Bivariate analyses were performed on Stata®14, significant *p* < 0.05.

**Findings:**

Global seroprevalence to the selected vector-borne pathogens was 74% (95%CI 65–83%). The highest seroprevalence was found for *E. canis/ewingii* (74%), followed by *A. phagocytophilum/platys* (16%). Seropositivity for *Borrelia* spp. and *Dirofilaria* spp. was 0%. All *Anaplasma* spp. seropositive dogs showed co-detection of *Ehrlichia* spp. (16%). Seroprevalence was significantly higher among dogs from families of lower socioeconomic status/level (I, 86%), followed by level II (74%), and III (36%) (*p* = 0.001). All dogs exhibiting anorexia (12%) were invariably seropositive (100%) (*p* = 0.029). Seroprevalence was higher among those showing mucocutaneous paleness (95%) compared to those without paleness (68%) (*p* = 0.013) (OR = 9.3; 95%CI 1.18–72.9). There was high variability in seroprevalence through the studied areas, ranging from 0% (La Libertad Park) up to Combia, Cesar Nader, Las Brisas and Saturno localities (100%) (*p* = 0.033).

**Interpretation:**

Given the high seroprevalence obtained in an area with documented ticks, there is a potential risk of zoonotic transmission to humans. Further seroprevalence studies in humans are needed to assess the prevalence of infections. Poverty is highly associated with these tick-borne pathogens in Pereira, as shown in the present study.

## Graphical abstract

**Figure:**
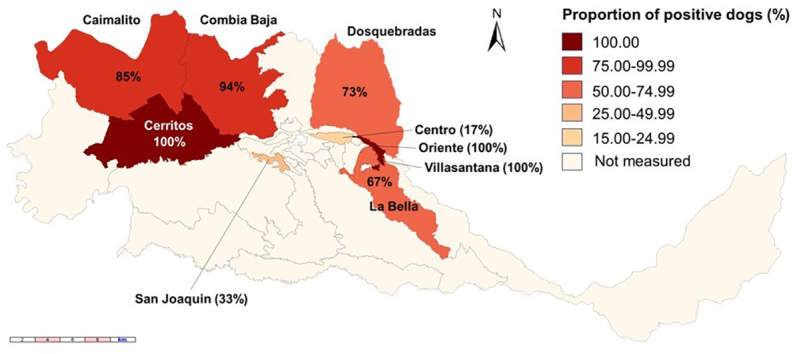

## 1 Introduction

Tick-borne diseases (TBD) are an essential group of infectious diseases that may affect both animals and humans, particularly in the tropical and subtropical regions of the world. Among these diseases is Lyme borreliosis, spotted fever group rickettsioses, Colorado tick fever, monocytic ehrlichiosis, tularemia, granulocytic anaplasmosis, among others (Alkishe et al., 2021; Karim et al., 2021; Negi et al., 2021).

Among the most vulnerable populations for these vector-borne diseases (Bonilla-Aldana et al., 2021) are dogs (Bonilla-Aldana et al., 2020b; Rodriguez-Morales et al., 2018a; Rodriguez-Morales et al., 2018b). Multiple studies worldwide have assessed the prevalence of such diseases in canine populations (Petruccelli et al., 2020). However, many countries still lack detailed studies, with many aspects concerning their epidemiology that remain yet to be clarified, including many societal aspects (Bayles and Allan, 2014; Peretti-Watel et al., 2019; Walker, 2011).

Unfortunately, TBD (such as ehrlichiosis, anaplasmosis), and dirofilariosis, are not reportable and currently not under surveillance in most Latin American countries (Baneth et al., 2020; Hernandez-Velasco et al., 2020; Mendes-de-Almeida et al., 2021; Polo et al., 2015; Robles et al., 2018; Wisely and Glass, 2019). In addition, there is a significant gap of knowledge about their circulation across endemic areas in certain countries, such as Colombia (Jaimes-Duenez et al., 2018; Miranda and Mattar, 2015; Pesapane et al., 2019; Santodomingo et al., 2019; Vargas-Hernandez et al., 2016). That is why seroprevalence studies remain crucial better to understand the diverse epidemiological aspects of these vector-borne diseases.

Herein, we present a prospective study aimed to evaluate the seroprevalence of *A. phagocytophilum/platys*‚ *Borrelia burgdorferi* sensu lato (s.l.), *E. canis/ewingii*, and *Dirofilaria immitis* infection and scrutinize the main associated factors related to the epidemiology of tick-borne diseases among dogs inhabiting the municipality of Pereira, Risaralda department, Colombia, in 2020.

## 2 Materials and methods

**Fig. 1:**
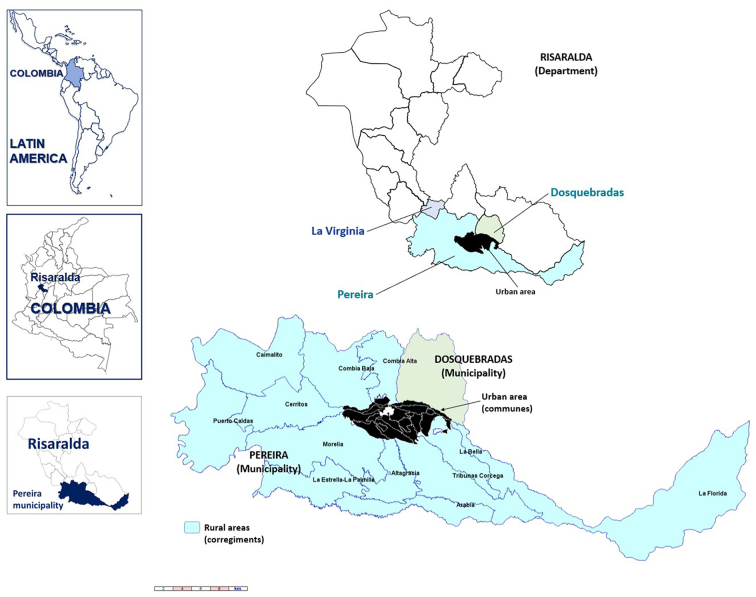
Relative location of Pereira and Dosquebradas municipalities, in the Risaralda department, Colombia, Latin America.

**Fig. 2:**
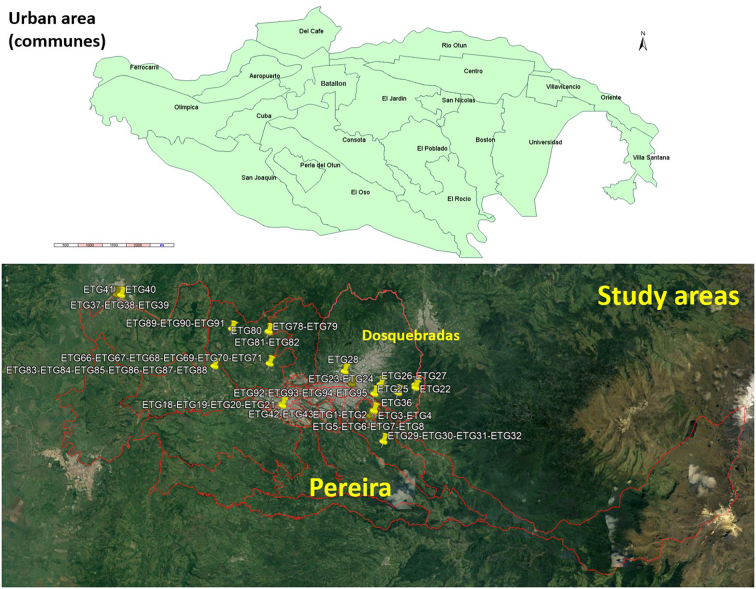
Urban areas (communes) of Pereira and the study areas in the metropolitan area (Pereira and Dosquebradas municipalities).

Pereira is the principal city of the Coffee-Triangle region, which includes three departments (first administrative territory level) and 53 municipalities (second administrative territory level). Pereira is the capital of Risaralda (967,780 habitants in 2020), a department surrounded by six other western departments (Antioquia, Caldas, Tolima, Quindio, Valle del Cauca and Chocó) (Rodriguez-Morales et al., 2015). Pereira's landscape embraces both urban and rural areas. The first consists of 20 communities (the city) and the second by 12 corregiments (sub-municipalities) (both tertiary administrative territory level) (Fig. 1). As per the National Statistics Department (DANE, www.dane.gov.co), the total population for 2020 reached 477,027 inhabitants. The metropolitan area includes Dosquebradas and La Virginia municipalities (Fig. 1), with 709,338 inhabitants for 2020. Rio Otún, Centro, San Joaquin, Del Café, Boston, El Oso, Consotá and Cuba, are the most populated areas of the Pereira municipality, making up 51% of its population (Fig. 2). The municipality of Pereira extends over an area of 702 Km^2^ (4°48′51″N 75°41′40″W). The climate is tropical, with an annual median temperature of 18.8 °C (median minimum of 15.8 °C, median maximum of 26.3 °C).

Based on the municipality dog's census, we calculated a minimum sample of 98 dogs to be assessed. Finally, 100 with infection suspicion were included distributed in four urban communes (out of 20) and four rural corregiments (out of 12) of the municipality Pereira and its neighboring municipality of Dosquebradas (Fig. 1). Only one dog was included from those owners who voluntarily accepted to participate with their dogs, regardless of where co-living was in the same house.

Blood samples from each dog were individually collected from the radial vein into a sterile vacuum tube (Vacutainer, Becton, Dickinson and Company Franklin Lakes, NJ, USA) without anticoagulant in the morning time. In the evening, samples were centrifuged at 1300–1800 ×*g* for 20 min, followed by serum separation from the clot.

To determine selected-vector-borne pathogens exposure, a rapid enzyme-linked immunosorbent assay (ELISA) kit (SNAP® 4Dx® Plus Test Kit, IDEXX Laboratories, Inc., Westbrook, ME, USA) was used following the manufacturer's instructions (Petruccelli et al., 2020). This qualitative test allowed us to simultaneously detect the presence of circulating antibodies (IgG and IgM) against immunodominant proteins of *Erhlichia canis/ewingii* (p30 and p30–1, sensitivity of 96.2%), *Anaplasma phagocytophilum/platys* (p44/MSP2, sensitivity of 99.1*%), Borrelia burgdorferi* s.l. (C6, the sensitivity of 98.8%), and *Dirofilaria immitis* antigens (principally produced by adult females) based on specific antibodies (sensitivity of 99.2%). The SNAP® 4Dx® Plus Test Kit showed a specificity of ~100% for the microorganisms mentioned above (Chandrashekar et al., 2010; Petruccelli et al., 2020; Stillman et al., 2014). 4Dx® Plus Test Kit has been validated in dogs (Chandrashekar et al., 2010; Petruccelli et al., 2020; Stillman et al., 2014).

Besides the general demographic, clinical and laboratory surveys, a questionnaire for social variables related to living conditions and households was also performed, including aspects such as: house vulnerability (defined according to five possible types of houses, from those with luxury and appropriate sanitary conditions to those without luxury and inappropriate sanitary conditions, last category considered as a vulnerable house); house location (in rural or urban areas); environmental elements close proximity to the house (e.g. small lakes, small rivers or wetlands); materials employed in the building of the walls (e.g. blocks, bricks, cement, wood, cardboard‑tin) and the floors (e.g. cement, wood, soil); access to tap water; need of water collection and its keeping at appropriate receptacles; and disposal of sewage water and waste disposal (e.g. by an urban service) (Quintero et al., 2012), among other social aspects such as the socioeconomic level of the house which is determined by the National Statistics Department (DANE).

Statistical analysis was performed using Stata 14®IC (Stata Corp., College Station, Texas, USA). Chi-square tests were used to compare proportions of positivity related to categorical dependent variables and establish statistical significance. In addition, Association between seropositivity and independent variables such as social house conditions, dog living conditions, and the socioeconomic level was evaluated. For all the independent variables, chi-square (χ^2^) and Fisher tests were used to assess associations and significance. In those significant values (*p* < 0.05), also the odds ratio with their 95% confidence interval (95%CI) was calculated.

The location of the houses hosting the dogs was georeferenced with their correspondent coordinates by the free mobile cell-GPS software application “Herramientas de GPS” v.3.1.0.5, developed by Virtual Maze® (www.virtualmaze.com). Seroprevalence was also presented by geographical information systems (GIS)-based maps. The georeferenced places were incorporated in the software Google Earth Pro®, and the layers of communes, corregiments and municipalities, provided by the Open Data Pereira Geographical Information System (https://mapas-pereira.opendata.arcgis.com/), in .kml files, were included to locate the coordinate's points in the corresponding shapes. Microsoft Access® software was used to design the spatial database to import the GIS software's incidence rates by corregiments and communas at Pereira municipality. The Client GIS software open source was Kosmo Desktop 3.0 RC1® (SAIG S.L., Madrid, Spain). For access to geographic data required and sharing results with institutions, support was provided by the spatial data infrastructure for the department by the Regional Information System of the Coffee-Triangle ecoregion (SIR) as standardized and reported before (Bonilla-Aldana et al., 2020a; Idarraga-Bedoya et al., 2020; Rodriguez-Morales et al., 2017). The shapefiles of corregiments and communas (.shp) were linked to a database through spatial joined operation to produce digital maps of the seroprevalence for the study area. Data used for the GIS-based maps were derived from the geographical origin of the serosurvey at the different geographic levels (Idarraga-Bedoya et al., 2020).

## 3 Results

The mean age of the canine population was 4.2 years (± 2.89 years, range 0.21–12.56), 53% were female, and 47% were males (Table 1). The average age did not differ significantly by sex (males 4.34 ± 3.27 years; females 4.10 ± 2.59 years) (*p* = 0.6345). Other dog variables are presented in Table 1. From the total number of canines, 89% presented clinical alterations, of them 100% presented anaemia, 54% lethargy, 39% alopecia, 21% decay, 21% mucocutaneous paleness, among others.

**Table 1:** Physical, clinical, and living conditions of the studied dogs.

Variable	Mean/n	SD/%	Minimum	Maximum
Age (years)	4.20	2.89	0.21	12.56
*Sex*				
Male	53	53.00		
Female	47	47.00		
Weight (kilograms)	13.99	7.76	4.00	40.00
*Body condition*				
1 (Very slim)	0	0.00		
2 (Slim)	21	21.00		
3 (Ideal)	50	50.00		
4 (Overweighted)	29	29.00		
5 (Obese)	0	0.00		
Body temperature (°C)	38.84	0.96	30.50	40.00
Cardiac rate (beats/min)	94.80	9.86	50.00	115.00
Respiratory rate (breaths/min)	30.83	4.51	24.00	55.00
*Capillary Refill Time (seconds)*				
1	0	0.00		
2	60	60.00		
3	28	28.00		
4	10	10.00		
5	2	2.00		
*Recent deworming (last month)*				
Yes	16	16.00		
No	84	84.00		
*Any vaccine (in the last year)*				
Yes	45	45.00		
*Rabies vaccine*	43	43.00		
No	55	55.00		
*Number of other dogs in the house*				
1	5	5.00		
2	13	13.00		
3	12	12.00		
>3	64	64.00		
*Number of cats in the house*				
0	32	32.0		
1	16	16.0		
2	9	9.0		
3	5	5.0		
>3	34	34.0		

The global seroprevalence to the selected vector-borne pathogens was 74% (95%CI 65–83%). The highest seroprevalence was found for *E. canis/ewingii* (74%), followed by *A. phagocytophilum/platys* (16%). Seropositive for *Borrelia* spp. and *Dirofilaria* spp. was 0%. All the *Anaplasma* spp. seropositive dogs were also positive for *Ehrlichia* spp. (16%).

Twenty-two per cent of the houses had rudimentary walls (adobe, cardboard, and palm), 84% rudimentary roofs (wood, zinc, and palms), 41% rudimentary floors (wood and earth), and 73% of the houses had only one bathroom. In those with only one bathroom, 100% had seropositive dogs, whilst in those houses with two or more bathrooms had 65% (*p* = 0.025). Although there was water supply (100%) in all the houses, 28% collected and store water (25% in tanks and 3% in plastic recipients). The water supply in 75% was by the public system, but the rest (25%) utilized water wells, rivers, water streams, and rainwater; 56% and the reported presence of rats in the house. Thirty-nine per cent of the dogs were allowed to be inside the house with the family; 10% of the dogs remained leashed in the house or the yard (all of them, 100%, were seropositive), whilst 90% were free to go anywhere (71% were seropositive) (χ^2^ = 3.903; *p* = 0.048); 82% of the dogs had access to wild areas. Regarding house waste, 81% was collected by the urban system, but the rest (29%) was burned. Illiteracy was reported in 2% of dog owners; 8% of dog owners were unemployed at the survey. Twelve per cent of owners lived in overcrowded houses (≥3 persons/room); in 70% of the owners, the number of persons per bathroom in their houses was ≥3.

**Fig. 3:**
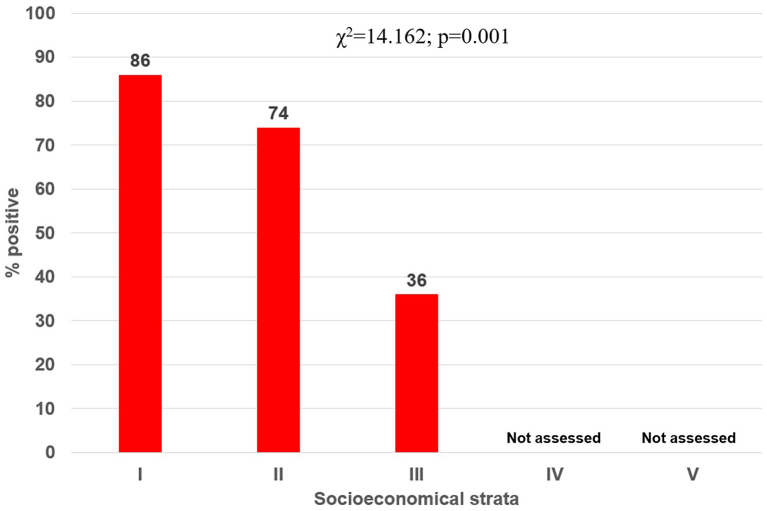
Seroprevalence by socioeconomic strata.

**Fig. 4:**
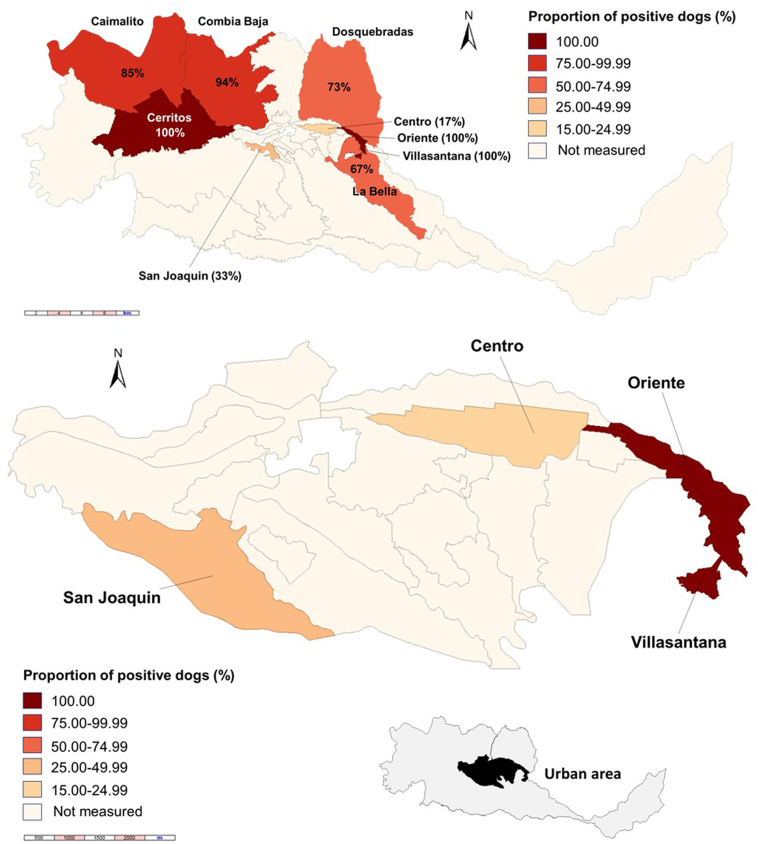
Seroprevalence in the municipalities of Pereira and Dosquebradas, metropolitan area, rural and urban areas of the capital of Risaralda.

The seroprevalence at rural (79%) and urban areas (67%) was not significantly different (*p* = 0.181). However, the seroprevalence was significantly higher in dogs from families of the lower socioeconomic level (I [Low-low, the lowest], 86%), followed by level II (74%), and III (36%) (χ^2^ = 14.162; *p* = 0.001); no dogs from the highest strata (IV and V) were available for our study (Fig. 3). There was high variability in seroprevalence by studied areas, ranging from 0% (La Libertad Park) up to Combia, Cesar Nader, Las Brisas and Saturno (100%) (*p* = 0.033). In the rural areas, Cerritos was the corregiment with the highest seroprevalence (100%), followed by Combia Baja and Caimalito (Fig. 4), whilst in the urban area, Oriente and Villasantana were the communes with the highest seroprevalence (100%) (Fig. 4).

All the dogs with anorexia (12%) were seropositive (100%) (χ^2^ = 4.791; *p* = 0.029). Seroprevalence was higher in those with mucocutaneous paleness (95%) compared to those without it (68%) (χ^2^ = 6.232; *p* = 0.013) (OR = 9.3; 95%CI 1.18–72.9).

## 4 Discussion

Poverty has been linked as a determinant to multiple disease groups, especially infectious diseases (Bukhman et al., 2020; Hummel et al., 2021; The Lancet Child Adolescent Health, 2019). Among the infectious diseases linked to poverty, tropical diseases and vector-borne diseases, such as malaria (Bi and Tong, 2014; Ricci, 2012; Santos-Vega et al., 2016; Tusting et al., 2016) and dengue (Bavia et al., 2020; Bonifay et al., 2017; Mulligan et al., 2015), have been well documented. Housing conditions are related to the occurrence and persistence of tropical infectious diseases (Cardona-Arias, 2018; Ercumen et al., 2019; Pham-Duc et al., 2013; Quintero et al., 2012; Snyman et al., 2015). There is a global lack of studies exploring the links between tick-borne diseases, such as *Ehrlichia* spp. infection, the primary pathogen that elicited antibodies in our study, and poverty. Poverty is also related to pet care as well as to tick control in dogs. In this study, none of the owners provided any veterinary care to their dogs over the last previous six months.

A recent study in Argentina, assessing different infectious diseases among dogs from a rural area in the humid Chaco, found 7.9% of them prevalent for ehrlichiosis. They suggest that their findings likely reflect structural poverty, poor sanitation and lack of a safe water supply (Enriquez et al., 2019). In another study from Brazil, assessing carthorses from low-income owners, authors, found that 27.4% were positive for *Ehrlichia* exposure (Vieira et al., 2016). However, none of these studies linked the pathogen prevalence to social factors or poverty directly and statistically. In our study, we observed a social profile of the studied population, but especially a significantly higher seroprevalence among those in the poor socioeconomic level, which is at the same time related to multiple other potentially associated factors that may be suitable for tick-borne and other tropical diseases.

This study was performed in Colombia, where there is also a lack of seroprevalence studies about *Ehrlichia* spp. (less than 20 in PubMed database), 11 in canine populations, none in Pereira and Risaralda (Arroyave et al., 2020b; Bonilla-Aldana et al., 2021; Forero-Becerra et al., 2021; Hidalgo et al., 2009; McCown et al., 2014; Miranda and Mattar, 2015; Pesapane et al., 2019; Vargas-Hernandez et al., 2012). Given the high seroprevalence obtained in an area with ticks' infestation (data not shown), the potential risk for zoonotic transmission to humans remains latent, deserving immediate seroprevalence studies.

Albeit their importance, emerging infectious diseases such as those caused by *Ehrlichia* and *Anaplasma* spp. are still neglected in many aspects that need to be understood, including clinical and epidemiological topics. Tick-borne diseases may lead to severe clinical consequences in animals and humans, leading to potentially fatal outcomes (Hardalo et al., 1995; Jahangir et al., 1998; Paddock et al., 1993; Tsiodras et al., 2017). Even for some species, there is still questioning whether these may cause, in addition, to dogs, infection in humans, such as is the case of *A. platys* (Arraga-Alvarado et al., 2014). Thus, more studies assessing their circulation in different hosts are needed to understand the ecoepidemiological associated factors, including the social ones, such as poverty (Godfrey and Randolph, 2011; Randolph, 2010; Sumilo et al., 2008). For example, in Eastern Europe, background socioeconomic conditions determine susceptibility to risk of tick-borne encephalitis; some authors suggested that increased unemployment during the economic recession of 2009 triggered a sudden increase in risk (Godfrey and Randolph, 2011). Additionally, regarding ticks, an ongoing assessment (unpublished data) has identified the presence of *Rhipicephalus sanguineus* sensu lato, in the area, then, this is expectable according the results of the current study. *R. sanguineus* sensu lato has a wide distribution in Colombia according to recent studies (Páez-Triana et al., 2021). In a recent study in Medellin, Colombia, with detection of *E. canis* and *A. platys* among dogs, *R. sanguineus* s.l. was the main tick identified (Arroyave et al., 2020a). Contrary, the presence of anthropophilic *Ixodes* ticks in Colombia is probably very low (Guglielmone et al., 2021), and even, only recently (2021), it was published the first report in the country of a species of the *Ixodes* genus parasitizing a human (Quintero et al., 2021). Then, as expected, the seroprevalence and circulation of *B. burgdorferi* s.l., is rare in Colombia, although a new putative taxon within the genus *Borrelia* has been detected from certain wild animals (e.g. bats) (Muñoz-Leal et al., 2021). Consistently, a previous study (2014) in Medellín, Barranquilla and Cartagena, also found 0% samples positive for *B. burgdorferi* s.l. in dogs (McCown et al., 2014). In the case of *Dirofilaria*, its prevalence in dogs has been found consistently low in different areas, e.g. 0% in Medellin, 2% in Barranquilla, 3% in Cartagena (McCown et al., 2014). In a recent study, its prevalence among dogs of Bucaramanga was 0.5% (by immunochromatography test kit) and 6.5% (by blood smears and modified Knott's test) (Muñoz et al., 2020).

Although we should acknowledge as a limitation that this study only assessed 100 dogs, preventing the possibility to run multivariate analyses, clearly evaluated multiple relevant social variables in addition to the clinical conditions of the animals, and the seroprevalence by a widely used (Kotwa et al., 2020; Mendes-de-Almeida et al., 2021; Petruccelli et al., 2020; Selim et al., 2021) serological diagnostic test for selected vector-borne pathogens in dogs, in different areas of the municipality, for the first also mapping, using geographical information system (GIS), the location of the assessed population, but particularly the distribution of the seropositive populations. Indeed, the use of GIS for mapping infectious diseases is highly valuable and previously used in our setting for human and animal diseases, such as malaria, Zika, neosporosis, among others (Bonilla-Aldana et al., 2020a; Idarraga-Bedoya et al., 2020; Rodriguez-Morales et al., 2015; Rodriguez-Morales et al., 2017). Its use, as shown here, would be helpful also in ehrlichiosis. These maps clearly showed the high seroprevalence in rural and poor communes in the urban area. The individual assessed conditions suggested that poverty is highly associated with these tick-borne pathogens in Pereira, as observed in this study.

Although *Ehrlichia chaffeensis*, *E. ewingii* and *A. phagocytophilum* are recognized as zoonotic agents, the role *E. canis* and *A. platys* as human pathogens is controversial (Ismail and McBride, 2017; Rar et al., 2021). Some reports have identified *E. canis* and *A. platys* in human samples (Arraga-Alvarado et al., 2014; Bouza-Mora et al., 2017; Perez et al., 2006). In the case of *A. phagocytophilum* this is the etiological agent of the human anaplasmosis (Price et al., 2022; Taank et al., 2020). In the case of *A. platys*, some reports suggest the possible human infection (Arraga-Alvarado et al., 2014). Then, there is a need for surveillance and study of human-dog interaction, assessing the potential for infections also in humans.

No previous studies on the epidemiology of tick-borne diseases and dirofilariasis in the city have been published so far. The current study was a cross-sectional, seroprevalence assessment, but further studies are expected.

## 5 Conclusions

Given the high seroprevalence obtained in an area with documented ticks, there is a potential risk of zoonotic transmission to humans. Further seroprevalence studies in humans are needed to assess the prevalence of infections. Poverty is highly associated with these tick-borne pathogens in Pereira, as shown in the present study.

## Ethics approval and consent to participate

All persons gave their informed consent before their inclusion in the study. Furthermore, according to standard protocols and guidelines from the Animal Ethics Committee (CICUA) at the Fundación Universitaria Autónoma de las Américas, Colombia, animal procedures were performed according to standard protocols and guidelines. Therefore, the CICUA approved and endorsed this study (Acta No. 27 de 2019).

## Consent for publication

All persons gave their informed consent before their inclusion in the study.

## Availability of data and materials

Available upon reasonable request.

## Funding

This study has been funded by Fundación Universitaria Autónoma de las Américas (UAM) (Schools of Veterinary Medicine and Zootechnics, and Medicine, Pereira), Universidad Nacional Autónoma de Honduras (10.13039/501100010301Faculty of Medical Sciences), Sci-Help, Vitalcare, IPS Cardiológica Eduardo Ramírez and Centro de Diagnóstico Especializado Testmol, RECEPA, the family Trejos-Mendoza, and the Dirección de Investigación Científica, Humanística y Tecnológica (DICIHT) (2-05-01-01). The current article processing charges (publication fees) were funded by the Facultad de Ciencias Médicas (FCM) (2-03-01-01), Universidad Nacional Autonoma de Honduras (UNAH), Tegucigalpa, MDC, Honduras, Central America (granted to Dr. Zambrano).

## Authors' contributions

DKBA conceived the investigation and supervised the field and laboratory work and data analyses (Conceptualization, Methodology, Project administration).

JPMA, MARM, AETM, SPV, LVM, LFMA, DON, MCP and EG, performed field and laboratory work (Data curation, Investigation); LVGC and AJRM revised and analyzed the clinical records of the patients and supervised laboratory work (Data curation, Investigation). SPV, AJRM and DKBA prepared the database with laboratory results and clinical data (Formal Analysis). AJRM and DKBA analyzed the data and wrote the first draft of the manuscript (Writing – original draft). All authors revised and approved the final version (Writing – review & editing).

## Declaration of Competing Interest

None.
